# The Frequency of Celiac Disease in Siblings of Celiac Patients and Applicability of Non-Biopsy Diagnostic Criteria

**DOI:** 10.3390/jcm15124740

**Published:** 2026-06-18

**Authors:** Ahmet Uzger, Ahmet Rauf Goktepe, Yasin Sahin

**Affiliations:** 1 Clinic of Pediatric Gastroenterology, Gaziantep City Hospital, Gaziantep 27470, Turkey; 2 Department of Pediatric Gastroenterology, Faculty of Medicine, Gaziantep Islam Science and Technology University, Gaziantep 27010, Turkey

**Keywords:** celiac disease, ESPGHAN, no-biopsy criteria, prevalence, siblings

## Abstract

**Objective:** First-degree relatives, especially siblings, are at increased risk of developing celiac disease (CD). The aim of this study was to determine the prevalence of CD in siblings of children with CD, and to evaluate the applicability of the non-biopsy diagnostic criteria recommended in the European Society for Pediatric Gastroenterology, Hepatology and Nutrition (ESPGHAN) 2020 guidelines. **Methods:** Siblings of children with biopsy-confirmed CD who had not previously been diagnosed with CD were included in the study. According to the ESPGHAN 2020 guidelines, cases with elevated anti-tTG IgA levels (more than 10 times the upper limit of normal) were diagnosed without biopsy. **Results:** In total, 250 siblings of 81 children with biopsy-confirmed CD were included in the study. The median age of the siblings was 9.00 years. Anti-tTG IgA positivity was detected in 31 siblings. A total of 26 siblings (10.4%) were diagnosed with CD. Of the diagnosed cases, 21 (80.76%) were asymptomatic. In 12 cases with anti-tTG IgA levels more than 10× ULN, the diagnosis was done without biopsy. **Conclusions:** The prevalence of CD in siblings of celiac patients was found to be 10.4%. This rate is approximately 22 times higher than in the general population in our country. Since half of the diagnosed patients are asymptomatic, screening all siblings for CD, regardless of the presence of symptoms, is crucial for early diagnosis. Additionally, diagnosis can be done without biopsy in eligible cases meeting the ESPGHAN 2020 no-biopsy criteria. **Note:** This article abstract has been accepted for the 58th ESPGHAN congress as an E-poster presentation.

## 1. Introduction

Celiac disease (CD) is a chronic, immune-mediated disorder triggered by the consumption of gluten and related prolamins in genetically predisposed individuals, characterized by small intestinal mucosal damage and a variety of clinical symptoms [1]. Its prevalence is estimated to be approximately 1% worldwide; however, this rate varies depending on geographical and genetic factors [1,2]. The prevalence of CD was reported to be 0.47% in a survey of healthy schoolchildren in Turkey [3].

Celiac disease has a multifactorial etiology, but genetic factors play a major role in its development. Human leukocyte antigen (HLA)-DQ2 and HLA-DQ8 haplotypes are the most significant genetic risk factors for CD [4,5]. These genotypes are present in about 30–40% of the general population, although 90–95% of celiac patients are HLA-DQ2-positive and the majority of the remaining individuals are HLA-DQ8-positive [4,6]. Due to the shared genetic predisposition within families, first-degree relatives of individuals diagnosed with CD, especially siblings, are at high risk of developing CD. It has been reported that the siblings have an 8–10 times higher prevalence of CD than the overall population, ranging from 3.9% to 11.9% [7,8,9,10,11,12].

In the past two decades, the clinical findings of CD have changed significantly. Classic malabsorption symptoms (chronic diarrhea, abdominal distension, growth retardation) are becoming less common, while extra-intestinal (iron deficiency anemia, elevated liver enzymes, short stature) or completely asymptomatic forms are being reported more frequently [13,14]. Therefore, early screening tests are important especially in high-risk groups. Early diagnosis followed by a strict gluten-free diet for life is crucial for preventing long-term complications such as osteopenia/osteoporosis, growth retardation, delayed puberty, infertility, and rarely lymphoma [15,16,17]. Therefore, both the European Society for Pediatric Gastroenterology, Hepatology and Nutrition (ESPGHAN) and the North American Society for Pediatric Gastroenterology, Hepatology and Nutrition (NASPGHAN) guidelines recommend screening first-degree relatives of patients diagnosed with CD, even if they are asymptomatic [4,6].

The ESPGHAN 2020 guidelines introduced a significant update allowing for the diagnosis of CD without duodenal biopsy in eligible cases that meet specific criteria [6]. This approach has the potential to reduce the physical and psychological stress, anesthesia risk, and cost associated with endoscopy, an invasive procedure. The aim of this study was to determine the prevalence of CD in siblings of children diagnosed with CD, to describe the clinical and demographic characteristics of diagnosed cases, and to evaluate the applicability of the ESPGHAN biopsy-free diagnostic criteria.

## 2. Materials and Methods

### 2.1. Study Population and Design

This retrospective, cross-sectional study was conducted in a tertiary pediatric gastroenterology clinic. The study included children with biopsy-confirmed CD, and their siblings aged 1–18 years who had not previously been diagnosed with CD.

Exclusion criteria:

- Being over 18 years of age,
- Having previously been diagnosed with CD,
- Having incomplete screening tests,
- Having additional autoimmune diseases that frequently co-occur with CD, such as type 1 diabetes mellitus and autoimmune thyroiditis.

The study protocol was approved by the local ethics committee (Gaziantep Islamic Science and Technology University Non-Interventional Ethics Committee, 19 November 2024/723.51.17). This study was conducted in accordance with the principles of the Helsinki Declaration. The Institutional Review Board waived informed consent of the included patients because the research involved analysis of retrospective data.

### 2.2. Screening and Diagnosis Algorithm

Serum anti-tissue transglutaminase IgA (anti-tTG IgA) (cut-off: 10 IU/L) and total IgA levels were measured in all siblings. Immunoturbidimetric method (Roche Diagnostics GmbH, Mannheim, Germany) was used for determination of total IgA and ELISA was performed in order to assess the tTG IgA (Aesku Diagnostics Gmbh, Wendelsheim, Germany). Only one kit was used during the study. The diagnostic approach was planned in accordance with the ESPGHAN 2020 guidelines [6]. Patients were divided into two groups:

**1.**  **group:** CD was diagnosed without a biopsy in eligible cases with anti-tTG IgA levels more than 10 times the upper limit normal (10× ULN) and positive anti-endomysium IgA (EMA) in a second blood sample.
**2.**  **group:** Upper gastrointestinal endoscopy was performed to confirm the diagnosis in cases with anti-tTG IgA levels less than 10× ULN, and multiple duodenal biopsies were obtained. All biopsy samples were evaluated according to the modified Marsh-Oberhuber classification [18]. Patients with Marsh stage 2 or 3 were diagnosed with CD.

### 2.3. Statistical Analysis

SPSS 25.0 (IBM Corp., Armonk, NY, USA) was used to analyze the data. For continuous variables with a normal distribution, descriptive statistics such as mean ± standard deviation, median (interquartile range) for non-normally distributed variables, and frequency (%) for categorical variables were used. Continuous variables between two independent groups were compared using either the Mann–Whitney U test or the independent samples t-test. The chi-square test was applied to compare categorical variables. Statistical significance was accepted as *p* < 0.05.

## 3. Results

The median age of the 250 siblings was 9.00 years (IQR: 5.10–12.52). In total, 129 of the patients were male (51.6%). The vast majority of siblings (96.0%) were asymptomatic. The median serum anti-tTG IgA level was found to be 0.69 IU/L (IQR: 0.30–1.49), and the median total IgA level was 107.70 mg/dL (IQR: 73.75–160.00) (Table 1). No IgA deficiency was detected in any patient.

Anti-tTG IgA positivity was detected in a total of 31 siblings (12.4%). Anti-endomysium IgA positivity was detected in the second blood sample of 12 cases in which anti-tTG IgA levels were ≥10× ULN (200 IU/L (IQR: 161.00–200.00) (Table 2). These 12 cases were diagnosed with CD without duodenal biopsy in accordance with ESPGHAN 2020 no-biopsy diagnostic criteria [6].

Of the 19 cases with positive anti-tTG IgA, the families of 5 refused endoscopy. The pathology results from all 14 cases were in line with Marsh stage 3. Then, CD was diagnosed in these patients. Anti-tTG IgA levels in this group were significantly lower than in the group diagnosed without biopsy (The median anti-tTG IgA levels: 18.98 IU/L, *p* < 0.001). Of the 250 siblings screened, 26 (10.4%) were diagnosed with CD. Of the 26 diagnosed cases, 21 (80.76%) were asymptomatic, and 5 (19.24%) were symptomatic.

The most common findings in the five symptomatic individuals were growth retardation (40.0%), recurrent abdominal pain (40.0%), and abdominal distention (20.0%).

## 4. Discussion

In the current study, the prevalence of CD in siblings of celiac patients was found to be 10.4%. This rate is approximately 22 times higher than in the general population in our country [3]. This marked difference may be attributed to the number of patients included during the cross-sectional study period and the single-center nature of the study.

It has been reported that the prevalence of CD among siblings is between 3.9% and 11.9% [7,8,9,10,11,12]. In the meta-analysis of Singh et al. [10], the pooled prevalence of CD among siblings was calculated as 8.9% among 10.252 first-degree relatives. The rate of 10.4% in our study is consistent with this meta-analysis and the upper limit values in the literature [9,10].

In a study of Vaquero et al. [19], histopathological abnormalities were detected in 48% of HLA-DQ2/8-positive adult first-degree relatives in Spain. Furthermore, the detection of mucosal atrophy in 6% of tTG-negative cases highlighted the limitations of serology-based screening in this study. In contrast to that, the histopathological confirmation performed on the vast majority of patients with serological positivity shows the effectiveness of our screening strategy. In our study, no seronegative cases had an endoscopy because no CD was thought to be present. However, since long-term follow-up of seronegative cases could not be performed, we do not know whether CD will develop in the future.

In studies conducted in our country, the prevalence of CD among siblings varies between 3.9% and 10.7% [5,8,20]. The higher prevalence reported in our study (10.4%) may be explained by the fact that the study was conducted in a tertiary care center and that the ESPGHAN 2020 non-biopsy diagnostic criteria were applied to all eligible patients. In fact, 12 siblings were diagnosed without biopsy in accordance with the ESPGHAN 2020 guideline. The larger sample size (*n* = 250) compared to previous studies allowed for a more precise calculation of prevalence. The fact that HLA-DQ2/DQ8 positivity was found in 90.2% of siblings in the study conducted by Sahin and Mermer [5] in our country supports the high genetic predisposition in our region.

It is noteworthy that 80.76% of diagnosed cases were asymptomatic. This situation demonstrates the prevalence of “silent” forms in the modern epidemiology of CD and that a diagnostic approach based solely on clinical suspicion will miss a significant portion of these individuals [13,14]. It has been reported that the rate of asymptomatic cases in first-degree relatives is between 28.4% and 45.2% [5,7,11,12,14,19,20]. The reason the asymptomatic rate is very high in our study may be the large number of cases, the cross-sectional nature of the study, the widespread use of family screening, and the regional differences.

Garampazzi et al. [13] suggested that the prevalence of asymptomatic forms in Italy increased to 10.6% in the last 5 years over a 20-year period, and that this increase was due to the widespread use of family screening. Our study’s higher prevalence could be attributed to both our location in a tertiary care facility and the widespread use of family screening.

Vaquero et al. [19] found no significant association between the presence of symptoms and the degree of duodenal damage in HLA-DQ2/8 positive relatives; they showed that serious mucosal damage can develop in asymptomatic individuals as well. The fact that 11 of the 14 cases diagnosed with CD by biopsy in our study were asymptomatic supports this view.

In asymptomatic individuals, ongoing immunological activity and chronic intestinal inflammation can lead to serious complications in the long term [15,16,17]. Some studies have shown that extraintestinal manifestations can be prevented with a gluten-free diet, while others have found that undiagnosed CD is a risk factor for refractory CD and enteropathy-associated T-cell lymphoma. Therefore, screening of high-risk groups, regardless of symptom presence, is vital for early diagnosis and prevention of complications.

In individuals with genetic predisposition, CD may undergo seroconversion over time [5,21]. Şahin and Mermer [5] reported that two siblings from the same family were diagnosed at different times (1 year and 4 years later). Goldberg et al. [21] reported that some family members who were initially seronegative became seropositive during follow-up. It was suggested that susceptibility should be confirmed by HLA-DQ2/DQ8 genotyping and that individuals who were initially negative should be serologically screened at regular intervals [5,21]. However, HLA-DQ2/DQ8 genotype analysis was not performed in our study, and long-term seronegative cases were not followed up. This is one of the limitations of our study.

According to the ESPGHAN 2020 guidelines, screening for CD in siblings is recommended at the time of diagnosis [6]. On the other hand, we performed serological screening tests for CD on the siblings of five celiac patients who had been diagnosed at another facility but the siblings had not undergone screening for CD despite being in a high-risk group. We diagnosed these five patients with CD according to the ESPGHAN 2020 guidelines. However, because they had not been screened within the timeframe recommended from the ESPGHAN guidelines, these five individuals experienced a diagnostic delay of 24 to 86 months.

In the present study, the most common findings in symptomatic cases diagnosed with CD were growth retardation (50%), recurrent abdominal pain (50.0%), and abdominal distension (20.0%). Over the last 10 years, a shift from classic symptoms to atypical symptoms has been observed in clinical findings [13]. Consistent with our study, a significant decrease in the prevalence of classic symptoms (from 76% to 44%) has been reported [13,14]. The findings of growth retardation in 50% and chronic abdominal pain in 50.0% of cases in our study indicate that the increase in atypical symptoms continues.

The current study highlights the clinical application of the ESPGHAN 2020 biopsy-free diagnostic approach within the specific context of sibling screening. In our cohort, two symptomatic cases were successfully managed without a biopsy, strictly adhering to the ESPGHAN 2020 guidelines. This approach effectively minimizes the risks, psychological stress, and financial costs associated with anesthesia and endoscopy for pediatric patients and their families [6]. While Werkstetter et al. [22] demonstrated that the non-biopsy approach offers comparable diagnostic accuracy to histological evaluation, our practical experience showed that patients with low-positive anti-tTG IgA titers still required a biopsy for definitive diagnosis. This underscores the clinical reality that histopathological confirmation remains indispensable for high-risk individuals with low antibody titers. In our cohort, all patients with low positive anti-tTG levels (<10× ULN) who underwent biopsy had histopathologically confirmed villous atrophy. However, this finding should not be interpreted in the context of the high pre-test probability of our study population and does not necessarily support lowering the biopsy threshold recommended by the ESPGHAN 2020 guidelines. Histopathological confirmation remains the gold standard in patients not fulfilling the non-biopsy criteria. Furthermore, while the ESPGHAN 2020 guidelines allow for a biopsy-free diagnosis regardless of symptoms, they strongly emphasize shared decision-making with families [6]. Following this recommendation, 12 eligible patients in our study received a biopsy-free diagnosis after comprehensive consultation with their families.

Previous literature supports the practice that patients with low-positive anti-tTG IgA levels and IgA deficiency should undergo biopsy confirmation [23]. Regarding asymptomatic individuals, Trovato et al. [24] identified severe histological damage (Marsh stage 3a-3c) in 92.5% of 40 asymptomatic patients with anti-tTG titers ≥ 10× ULN, suggesting that the degree of mucosal injury in asymptomatic cases with high titers mirrors that of symptomatic patients. The outcomes of our study reflect these observations, demonstrating the feasibility of the biopsy-free pathway in asymptomatic screening cohorts with high anti-tTG titers, while concurrently illustrating the clinical necessity of biopsies in cases with low positive anti-tTG IgA levels [6,23]. Ultimately, our implementation shows that the ESPGHAN criteria can be safely and effectively applied to select asymptomatic high-risk individuals, as demonstrated by the 12 out of 26 patients successfully diagnosed without an invasive procedure.

The strengths of our study were conception and execution in accordance with the current ESPGHAN 2020 diagnostic criteria, and diagnosis of CD without biopsy in eligible patients.

The limitations of our study include its single-center nature, the lack of HLA genotype analysis, and the absence of long-term follow-up for seronegative cases. The fact that the families of 5 out of 19 cases who had positive celiac antibodies refused endoscopy may have led to a lower prevalence than it actually is.

## 5. Conclusions

This study showed that siblings of children diagnosed with CD have a high risk of the disease (10.4%) and that a significant proportion of cases (80.76%) may be asymptomatic. Furthermore, our findings suggest that the ESPGHAN 2020 non-biopsy criteria may be applicable in selected high-risk cases in routine clinical practice.

As a result, since siblings of patients with CD have a very high risk of developing the disease, serological screening tests should be performed on siblings at the time of admission regardless of symptom status. Long-term complications can be prevented with early diagnosis and treatment.

## Figures and Tables

**Table 1:** The demographic characteristics of the patients.

Variables	Value
Age (years) *	9.00 (IQR: 5.10–12.52)
Sex (m/f)	129/121
Height (cm) ^#^	126.81 ± 25.86
Weight (kg) ^#^	30.14 ± 16.48
tTG-IgA (IU/L) *	0.69 (IQR: 0.30–1.49)
total IgA value (mg/dL) *	107.70 (IQR: 73.75–160.00)

* Data are presented as median (IQR: Q1–Q3). ^#^ Data are presented as mean ± SD.

**Table 2:** The comparison of two groups in terms of demographic and laboratory features.

Variables	1. Group (*n* = 12)	2. Group (*n* = 14)	*p*
Age (years) *	8.05 (IQR: 5.07–11.27)	8.50 (IQR: 3.00–11.52)	0.940
Sex (m/f)	6/6	4/10	0.400
Height (cm) ^#^	137.00 ± 19.78	126.93 ± 38.51	0.742
Weight (kg) ^#^	31.14 ± 12.02	32.26 ± 15.95	0.940
Median diagnostic			
delay (months) *	2.50 (IQR: 1.62–4.75)	3.50 (IQR: 2.00–24.00)	0.742
tTG-IgA (IU/L) *	200 (IQR: 161.00–200.00)	18.98 (IQR: 13.08–95.25)	0.001
total IgA value (mg/dL) ^#^	140.33 ± 50.93	151.87 ± 65.42	0.527

* Data are presented as median (IQR: Q1–Q3). ^#^ Data are presented as mean ± SD.

## Data Availability

The datasets used and/or analysed during the current study are available from the corresponding author on reasonable request.

## Abbreviations

The following abbreviations are used in this manuscript:

CD	Celiac disease
ESPGHAN	European Society of Pediatric Gastroenterology, Hepatology and Nutrition
HLA	Human leukocyte antigen
Anti-tTG	Anti-tissue transglutaminase
